# Impact of mitochondrial metabolism on T-cell dysfunction in chronic lymphocytic leukemia

**DOI:** 10.3389/fcell.2025.1577081

**Published:** 2025-04-17

**Authors:** Wael Gamal, Melanie Mediavilla-Varela, Vishaal Kunta, Eva Sahakian, Javier Pinilla-Ibarz

**Affiliations:** ^1^ Department of Immunology, H. Lee Moffitt Cancer Center and Research Institute, Tampa, FL, United States; ^2^ Department of Malignant Hematology, H. Lee Moffitt Cancer Center and Research Institute, Tampa, FL, United States

**Keywords:** CLL (chronic lymphocytic leukemia), T-cell exhaustion, mitochondria, CAR T cell, cancer, adoptive cell immunotherapy, metabolism

## Abstract

T cells play a central role in anti-tumor immunity, yet their function is often compromised within the immunosuppressive tumor microenvironment, leading to cancer progression and resistance to immunotherapies. T-cell activation and differentiation require dynamic metabolic shifts, with mitochondrial metabolism playing a crucial role in sustaining their function. Research in cancer immunometabolism has revealed key mitochondrial abnormalities in tumor-infiltrating lymphocytes, including reduced mitochondrial capacity, depolarization, structural defects, and elevated reactive oxygen species. While these mitochondrial disruptions are well-characterized in solid tumors and linked to T-cell exhaustion, their impact on T-cell immunity in lymphoproliferative disorders remains underexplored. Chronic lymphocytic leukemia (CLL), the most prevalent chronic adult leukemia, is marked by profound T-cell dysfunction that limits the success of adoptive cell therapies. Emerging studies are shedding light on the role of mitochondrial disturbances in CLL-related T-cell dysfunction, but significant knowledge gaps remain. This review explores mitochondrial metabolism in T-cell exhaustion, emphasizing recent findings in CLL. We also discuss therapeutic strategies to restore T-cell mitochondrial function and identify key research gaps.

## Introduction

The concept of cancer immunosurveillance was introduced in the 20^th^ century, referring to the ability of the immune system to detect and eliminate cancer cells (Burnet and Schwartz, 1970; Penn and Starzl, 1970; Ross Sheil, 1986). This theory paved the way for cancer immunotherapy, leveraging adaptive immune cells—particularly T cells, the primary components of the adaptive immune system—to target and destroy cancer cells. As a result, various T-cell therapies have been developed. One example is immune checkpoint blockade therapy (Sharma and Allison, 2015), which restores T-cell anti-tumor function by overcoming immune suppression. Other approaches include adoptive T-cell therapies such as chimeric antigen receptor (CAR) T-cell therapy and tumor-infiltrating lymphocyte (TIL) therapy (Porter et al., 2011; Dudley et al., 2002).

T-cell development begins with lymphocyte progenitors in the bone marrow, which migrate to the thymus to undergo maturation and selection. The two main subtypes of mature T cells are CD4^+^ T helper cells and CD8^+^ cytotoxic T cells, which play a central role in direct anti-tumor responses. Naïve T-cell priming occurs in the draining lymph nodes where processed tumor-derived antigens are presented by antigen-presenting cells, particularly dendritic cells. The presented antigens interact with the T-cell receptor (TCR)-CD3 complex on T cells via the major histocompatibility complex (MHC) molecules (Chen and Mellman, 2013). This interaction and secondary signals from costimulatory molecules such as CD28 and cytokines like IL-2, IL-7, and IL-15 lead to tumor-specific T cells' activation and clonal expansion. Activated T cells traffic through the blood and lymphatic systems to the tumor site, infiltrating the tumor bed and recognizing cancer cells through their tumor-specific antigens. CD8^+^ cytotoxic T cells then kill tumor cells by releasing effector molecules such as granzymes and perforin, which induce apoptosis, as well as pro-inflammatory cytokines like IFN-γ and TNF-α (Chen and Mellman, 2013).

**FIGURE 1:**
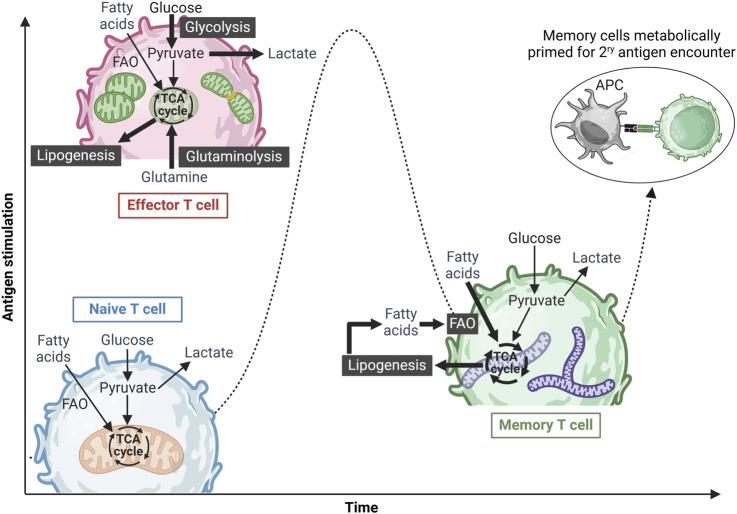
Metabolic reprogramming of T cells upon antigen stimulation. Naïve T cells exist in a metabolically quiescent state, primarily utilizing basal oxidative phosphorylation (OXPHOS) to meet their minimal energy demands. Upon antigen recognition and activation, these cells differentiate into effector T cells, undergoing a metabolic shift toward an anabolic state characterized by increased aerobic glycolysis, glutamine oxidation, and OXPHOS to support rapid expansion and effector functions. Effector T cells also exhibit mitochondrial fission, mediated by Drp-1, to accommodate heightened metabolic demands. Following antigen clearance and resolution of the immune response, a subset of surviving T cells differentiates into memory T cells, reverting to a metabolically quiescent state predominantly reliant on OXPHOS and fatty acid oxidation (FAO) for long-term survival and energy homeostasis. Memory T cells can also utilize glucose-derived ATP to synthesize fatty acids, which are subsequently broken down via FAO, a process essential for their long-term maintenance. Additionally, these cells are characterized by fused mitochondrial networks with high spare respiratory capacity (SRC), enabling them to rapidly respond to subsequent antigenic challenges. Figure was created in BioRender. Gamal et al. (2025), https://BioRender.com/p2qlxtp.

T-cell activation and differentiation processes are energy-intensive and driven by significant metabolic shifts, with mitochondria playing a key role. Figure 1 summarizes these metabolic changes. Naive T cells sustain their low energy demands primarily through oxidative phosphorylation (OXPHOS) within the mitochondria. They also catabolize fatty acids and amino acids to fuel the tricarboxylic acid (TCA) cycle for adenosine triphosphate (ATP) production (Delgoffe and Powell, 2015). Upon activation, T cells shift to an anabolic metabolism, relying on aerobic glycolysis (the Warburg effect (Warburg, 1956)) and OXPHOS to support the rapid proliferation and effector functions (Chang et al., 2013). During this metabolic shift, amino acids support protein synthesis, and fatty acids aid in membrane formation. After the immune challenge is resolved, surviving memory T cells acquire a quiescent state but remain metabolically primed for persistence and rapid reactivation. Compared to naive and effector cells, memory T cells exhibit higher mitochondrial mass, and rely on fatty acid oxidation (FAO) and OXPHOS. They also possess elevated mitochondrial spare respiratory capacity (SRC), ensuring sustained energy availability for future immune responses (van der Windt and Pearce, 2012; O’Sullivan et al., 2014; Pearce et al., 2009; van der Windt Gerritje et al., 2012).

As shown in Figure 1, mitochondrial morphology undergoes dynamic changes during T-cell activation (van der Windt Gerritje et al., 2012; Buck Michael et al., 2016), playing a key role in electron transport chain (ETC) complex formation and ATP production via OXPHOS (Buck Michael et al., 2016). Memory T cells maintain more elongated mitochondrial networks than effector T cells, enabling efficient FAO and elevated SRC (Buck Michael et al., 2016; Hermans et al., 2020; Bantug et al., 2018; Simula et al., 2018). Studies have shown that the mitochondrial dynamin-like GTPase (OPA1), which regulates mitochondrial cristae structure and ETC function (Cogliati et al., 2013), is crucial for central memory CD8^+^ T-cell development (Buck Michael et al., 2016). Similarly, losing Drp1, a mitochondrial fission protein, leads to elongated fused mitochondria, favoring a memory T-cell phenotype (Simula et al., 2018). Furthermore, evidence has shown that Drp1-mediated mitochondrial fission at the immunological synapse and the intracellular mitochondrial positioning are essential for optimal CD8^+^ T-cell activation (Baixauli et al., 2011). However, it is crucial to cautiously interpret the role of mitochondrial fission/fusion and FAO in memory T-cell development. Recent studies have shown that Mdivi-1, commonly used as a specific mitochondrial fission inhibitor, is not a selective Drp1 inhibitor (Bordt et al., 2017). Similarly, etomoxir, widely used to block mitochondrial long-chain FAO, has been found to affect T-cell differentiation independently of Cpt1a expression, the key enzyme regulating long chain-FAO (Raud et al., 2018).

Therefore, mitochondrial integrity is essential for T-cell anti-tumor activity. The metabolically harsh and nutrient-deprived tumor microenvironment (TME) can severely disrupt T-cell mitochondrial function, contributing to cancer progression and immune evasion (Yu et al., 2020; Scharping et al., 2016). In the context of adoptive T-cell therapy (ACT), mitochondrial disturbance is emerging as a key mechanism of therapy resistance, which might limit the number of patients responding to this promising immunotherapy (Rostamian et al., 2021).

Chronic lymphocytic leukemia (CLL) is a clonal B-cell malignancy, primarily impacting the elderly populations in Western societies (Hallek, 2019; Wierda et al., 2019). The treatment landscape for CLL has seen groundbreaking changes with the introduction of targeted therapies, specifically Bruton’s tyrosine kinase (BTK) and B-cell lymphoma 2 (Bcl-2) inhibitors (Herman et al., 2011; Souers et al., 2013; Herman et al., 2010). Nonetheless, such therapies are not considered curative and require prolonged periods of treatment, which evokes acquired mutations and resistance (Woyach et al., 2014). Autologous T-cell-based immunotherapies, particularly CAR T cells, have exhibited substantial success, resulting in multiple approvals by the FDA for various hematological malignancies (Khan et al., 2024; Grover et al., 2022). However, the success rate of CAR T-cell therapy in relapsed/refractory CLL patients is notably limited (20% complete response (CR) rate and, therefore, even fewer long-term remissions) (Siddiqi et al., 2023). This limitation is attributed to the acquired dysfunctional nature of T cells in CLL, which exhibit skewing towards immunosuppressive and terminally differentiated phenotypes (Forconi and Moss, 2015; Görgün et al., 2005; Riches et al., 2013; Fraietta et al., 2018; Gamal et al., 2023).

Recent research has explored the involvement of mitochondrial dysfunction in CLL T-cell impairment, aiming to uncover novel molecular mechanisms (Gamal et al., 2025; van Bruggen et al., 2019; van Bruggen et al., 2022). This review examines how disruptions in mitochondrial metabolism within the TME contribute to T-cell exhaustion and immune evasion, focusing on recent advances in CLL. We broadly discuss emerging strategies to restore or reprogram mitochondrial function to enhance T-cell immunity and ACT in CLL and cancer.

## Mitochondrial disturbance and T-cell exhaustion

The term “T-cell exhaustion” refers to the progressive loss of cytolytic activity and diminished responsiveness of CD8^+^ T cells within the TME and during chronic infections (Wherry and Kurachi, 2015; Ghoneim et al., 2017; Baitsch et al., 2011; Abdel-Hakeem et al., 2021). Chronic antigenic stimulation and immune-suppressive cells and mediators in the TME drive this process, leading to cancer immune evasion. Exhausted T cells are characterized by the persistent expression of multiple inhibitory receptors or checkpoint markers, including PD-1, Lag-3, Tim-3, and CTLA-4, which suppress T-cell activity (Wherry and Kurachi, 2015). Beyond checkpoint expression, T-cell exhaustion follows a distinct differentiation trajectory, marked by unique transcriptional and epigenetic signatures that distinguish them from short-lived effector cells and memory T cells (Abdel-Hakeem et al., 2021; Beltra et al., 2020). Exhausted T cells are heterogenous, consisting of diverse subpopulations that develop from precursor cells. This process is regulated by transcription factors such as TOX, Eomes, T-bet, and TCF-1 (Beltra et al., 2020). Exhausted T-cell subsets vary in their effector function, proliferative capacity, and responsiveness to immunotherapy, which influence therapeutic outcomes in cancer treatment (Rahim et al., 2023; Siddiqui et al., 2019).

The TME presents a metabolically hostile niche marked by nutrient deprivation, including excessive glucose consumption by tumor cells and depletion of key amino acids such as arginine and tryptophan. These metabolic constraints profoundly affect T-cell function, driving T-cell exhaustion in cancer (Franco et al., 2020; Chang et al., 2015). Emerging evidence highlights mitochondrial dysfunction as a critical factor in this process, which impairs T-cell fitness and anti-tumor immunity. CD8^+^ tumor-infiltrating lymphocytes (TILs) exhibit abnormal mitochondrial morphology, diminished activity, and impaired biogenesis (Scharping et al., 2016). This dysfunction is primarily due to reduced expression of peroxisome proliferator-activated receptor gamma coactivator 1-alpha (PGC1α), a key regulator of mitochondrial biogenesis and antioxidant responses. Additionally, CD8^+^ T cells within the TME and chronic infection models display depolarized mitochondria, characterized by reduced mitochondrial membrane polarization relative to mitochondrial mass, along with impaired glucose uptake—features closely associated with T-cell exhaustion and diminished effector function (Yu et al., 2020; Scharping et al., 2016; Bengsch et al., 2016; Schurich et al., 2016). Mitochondrial membrane depolarization in TILs correlates with structural alterations, including disrupted cristae organization and reduced cristae number and length per mitochondrion (Yu et al., 2020). These findings suggest that mitochondrial adaptation may attempt to compensate for impaired function. Furthermore, the accumulation of depolarized mitochondria is strongly linked to elevated mitochondrial reactive oxygen species (ROS) levels (Yu et al., 2020; Fisicaro et al., 2017; Siska et al., 2017). While mitochondrial ROS play a crucial role in normal T-cell activation (Sena Laura et al., 2013; Kamiński et al., 2010), excessive ROS accumulation promotes T-cell exhaustion through persistent activation of nuclear factor of activated T cells (NFAT) signaling (Scharping et al., 2021; Reina-Campos et al., 2021).

Hypoxia, coupled with nutrient deprivation, severely impairs T-cell function in cancer. Increased oxygen consumption by tumors has been associated with T-cell exhaustion and resistance to PD-1 blockade therapy (Najjar et al., 2019). Prolonged T-cell stimulation under hypoxic conditions exacerbates dysfunction by inducing mitochondrial impairment and excessive ROS accumulation (Scharping et al., 2021). Recent findings indicate that hypoxia-inducible factor 1-alpha (HIF-1α) plays a key role in T-cell dysfunction during chronic antigenic stimulation. It drives the transition of precursor-exhausted T cells to terminally exhausted cells in response to mitochondrial dysfunction and oxidative stress (Wu et al., 2023).

Epigenetic regulation of gene expression is crucial for the development and persistence of effector function in CD8^+^ T cells, while also influencing their stemness and fate (Henning et al., 2018; Dai et al., 2021). Exhausted T cells exhibit a unique epigenetic signature that sets them apart from effector and memory cells (Belk et al., 2022). Recent evidence suggests that impaired metabolic and mitochondrial function can alter the balance of key intermediate metabolites, influencing epigenetic reprogramming (Franco et al., 2020; Matilainen et al., 2017). For instance, acetyl-CoA regulates histone acetylation at specific loci, including *Ifng* and *Tbx21* (Peng et al., 2016; Qiu et al., 2019). Similarly, S-adenosyl methionine (SAM), a vital methyl donor for DNA and histone methylation, is influenced by α-ketoglutarate (α-KG)-mediated demethylation, which can play a role in T-cell exhaustion in cancer (Ye and Tu, 2018; Nabe et al., 2018). Additionally, lactate produced via aerobic glycolysis has been linked to a novel histone modification, lactylation, which enhances gene transcription and may contribute to the impairment of the anti-tumor immune response (Zhang et al., 2019; Peralta et al., 2024; Ma et al., 2024). This intricate crosstalk between metabolism and epigenetics provides critical insights into T-cell exhaustion, particularly within the TME.

Recent advances in understanding cancer immune evasion have revealed that immune checkpoint interactions are not the sole mechanism driving immune resistance. This offers insights into why immune checkpoint therapies fail in a substantial subset of cancer patients. Cutting-edge microscopy, mitochondrial tracing, and functional studies have uncovered a novel mechanism by which cancer cells hijack mitochondria from T cells through intercellular nanotube structures. This mitochondrial transfer enhances the mitochondrial function and proliferation of cancer cells while compromising T-cell mitochondrial fitness (Saha et al., 2022).

Zhang et al. developed a computational method named MERCI (mitochondrial-enabled reconstruction of cellular interactions) to investigate this phenomenon in human tumors. This method infers mitochondrial transfer from T cells to cancer cells by integrating mitochondrial mutation and gene expression data from single-cell RNA sequencing (scRNA-seq) of patient samples (Zhang et al., 2023). This approach revealed novel cancer phenotypes linked to mitochondrial transfer in human tumor samples. Analysis of patient samples further indicated that this process of mitochondrial transfer can predict adverse clinical outcomes, potentially via the enhancement of cancer cell proliferation.

More recently, studies sequencing mitochondrial DNA (mtDNA) in TILs identified shared mutations with cancer cells (Ikeda et al., 2025). Interestingly, the transfer of mtDNA-mutated mitochondria from cancer cells to T cells in the TME contributed to T-cell dysfunction. Notably, the presence of mtDNA mutations in tumor samples from patients with melanoma and non-small cell lung cancer correlated with poor survival responses to PD-1 blockade therapy (Ikeda et al., 2025). These findings highlight that mitochondrial transfer is considered a novel key driver of T-cell impairment and immune evasion in cancer.

## T-cell dysfunction in CLL

T-cell exhaustion is well-established in solid tumors and chronic viral infections. However, it is less understood in hematological malignancies due to the distinct nature of the TME in these cancers. T-cell therapies, particularly CAR T cells, have achieved significant success in hematological malignancies such as relapsed/refractory diffuse large B-cell lymphoma (DLBCL) and acute lymphoblastic leukemia (ALL) (Khan et al., 2024; Grover et al., 2022). However, it was not until March 2024 that the FDA granted accelerated approval to lisocabtagene maraleucel as the first CAR T-cell therapy for relapsed/refractory CLL patients. Despite that, the results from the pivotal TRANSCEND CLL 004 trial reported a complete response rate of only 20% (Siddiqi et al., 2023), highlighting a significant limitation in CAR T-cell efficacy for CLL. This suboptimal response is primarily attributed to the dysfunctional phenotype of the patient-derived T cells used in CAR T-cell manufacturing.

CLL is characterized by the clonal expansion of mature CD19^+^CD5^+^CD23^+^CD20^+^ B lymphocytes, found in peripheral blood, bone marrow, and secondary lymphoid organs such as the spleen and lymph nodes (Hallek, 2019; Wierda et al., 2019). CLL patients may present with cytopenias, lymphadenopathy, hepatomegaly, and splenomegaly. While B-cell receptor signaling plays a critical role in CLL pathogenesis, the survival and proliferation of CLL cells are largely dependent on signals from the microenvironment within secondary lymphoid organs—key sites of CLL proliferation (Herndon et al., 2017; Koehrer and Burger, 2023). This includes interactions with bone marrow stromal cells, monocyte-derived nurse-like cells (NLCs), and T cells, collectively shaping disease progression and therapeutic resistance.

The role of T cells in CLL remains a subject of active investigation, with evidence suggesting both pro- and anti-leukemic functions (Roessner and Seiffert, 2020; Vlachonikola et al., 2021). Numerous studies have demonstrated that T-cell differentiation in CLL is skewed toward effector and/or terminally differentiated phenotypes (Catovsky et al., 1974; Tötterman et al., 1989; Christopoulos et al., 2011; Palma et al., 2017; Elston et al., 2020). Significant efforts have been made to characterize the function and predominant phenotype of CD4^+^ T-helper (Th) cells in CLL. A predominant Th2 phenotype has been observed in peripheral blood mononuclear cells (PBMCs) from CLL patients and in the Eμ-TCL1 murine model, the most widely used preclinical model for CLL. This phenotype skewing has been linked to dysregulated gene expression in pathways such as the mitogen-activated protein kinase (MAPK) and the c-JUN N-terminal kinase signaling, contributing to CLL progression (Görgün et al., 2005; Podhorecka et al., 2002; Rossmann et al., 2002; McClanahan et al., 2015). On the contrary, other studies have reported increased Th1 cells producing IFNγ in CLL patients and Eμ-TCL1 mice, suggesting a role in promoting CLL-cell activation (Podhorecka et al., 2002; Os et al., 2013; Roessner et al., 2020; Bürgler et al., 2015; Zaki et al., 2000). Follicular Th cells (Tfh), which typically support B-cell activation within germinal centers of lymphoid tissues, were recorded to be elevated in the blood and lymph nodes of CLL patients, where they support disease progression through IL-21 cytokine production (Ahearne et al., 2013; de Weerdt et al., 2019; Cha et al., 2013; Pascutti et al., 2013; Vaca et al., 2022). Furthermore, regulatory T cells (Tregs), recognized for their immunosuppressive and pro-tumorigenic functions (Takeuchi and Nishikawa, 2016), are elevated in the peripheral blood of CLL patients and linked to poor prognosis (Lad et al., 2013; Giannopoulos et al., 2008; Biancotto et al., 2012; D’Arena et al., 2011; Piper et al., 2011). Tregs in CLL exhibit high expression of immunosuppressive markers such as CTLA-4 and secrete cytokines like IL-10 and TGF-β, further dampening anti-tumor immunity (Motta et al., 2005; Wierz et al., 2018; Beyer et al., 2005; De Matteis et al., 2018). Another distinct subset of CD4^+^ Th cells, Th17 cells—primarily involved in immune homeostasis and defense against extracellular pathogens (Steinman, 2007; Ouyang et al., 2008)—were also found to be more abundant in the peripheral blood of CLL patients. Interestingly, increased Th17 cell levels have been associated with improved clinical outcomes (Gamal et al., 2023; Hus et al., 2013; Jadidi-Niaragh et al., 2013), suggesting a potential protective role in CLL.

CD8^+^ T cells exhibit altered differentiation in CLL, favoring antigen-experienced effector and effector memory phenotypes over naïve and central memory phenotypes seen in age-matched healthy donors (Riches et al., 2013; Gamal et al., 2025; McClanahan et al., 2015; Hanna et al., 2019; Tonino et al., 2012; Mackus et al., 2003; Brusa et al., 2013; Göthert et al., 2013; Gonnord et al., 2019). This shift is marked by simultaneous increase in inhibitory receptors (e.g., PD-1, CD244, CD160) and functional cytotoxic molecules (e.g., IFNɣ, GZMb) (Riches et al., 2013; Gamal et al., 2025; McClanahan et al., 2015; Hanna et al., 2019). Research has highlighted the potential anti-leukemic role of CD8^+^ T cells in CLL, demonstrated by clonal expansion in patients and murine models (Hanna et al., 2019; Vardi et al., 2016; Vardi et al., 2017; Blanco et al., 2018; Serrano et al., 1997; Kowalewski et al., 2015). Furthermore, adoptive transfer experiments using the Eμ-TCL1 murine model have shown that CD8^+^ T cells can effectively suppress leukemia progression and extend survival in leukemic mice (Hanna et al., 2019).

However, despite their anti-leukemic potential, CD8^+^ T cells in CLL develop functional impairments that hinder their efficacy. These defects include diminished proliferative capacity and weakened cytolytic activity upon *ex vivo* activation (Riches et al., 2013). Gene expression profiling of CD8^+^ T cells from CLL patients has uncovered disruptions in key cellular processes such as actin polymerization, vesicle trafficking, and cytotoxicity signaling pathways (Görgün et al., 2005), reinforcing the concept of T-cell dysfunction in CLL. Additionally, studies have reported defective immune synapse formation between T cells and CLL cells, further compromising their function (Ramsay et al., 2008; Gorgun et al., 2009; Ramsay et al., 2012).

The extent to which CD8^+^ T-cell dysfunction in CLL parallels the classical exhaustion phenotype seen in solid tumors and chronic infections remains unresolved. While CLL-derived CD8^+^ T cells express high levels of PD-1 and other inhibitory receptors, they retain cytokine-producing abilities (Riches et al., 2013; Hanna et al., 2019), leading to their classification as “pseudoexhausted” (Riches et al., 2013). Notably, comparative analyses of CD8^+^ T cells from peripheral blood and lymph nodes in CLL patients suggest that exhaustion features are more pronounced in the lymph nodes (de Weerdt et al., 2019; Hanna et al., 2019).

Recent findings by Hanna et al. have further refined our understanding of CD8^+^ T-cell heterogeneity in CLL, identifying two distinct subpopulations. The first consists of PD-1^hi^ cells with functional impairment while the second includes PD-1^int^TCF-1^+^ cells, which resemble progenitor exhausted T cells with retained functionality, akin to those found in chronic infections (Hanna et al., 2021). While CD8^+^ T cells in CLL can recognize tumor-specific antigens, ongoing debate persists regarding whether their dysfunction is driven by chronic antigenic stimulation. Intriguingly, recent murine studies indicate that CLL cells may also induce dysfunction in bystander T cells (Martens et al., 2023), adding another layer of complexity to the mechanisms underlying CD8^+^ T-cell impairment in CLL.

## Mitochondrial dysregulation and T-cell activity in CLL

**FIGURE 2:**
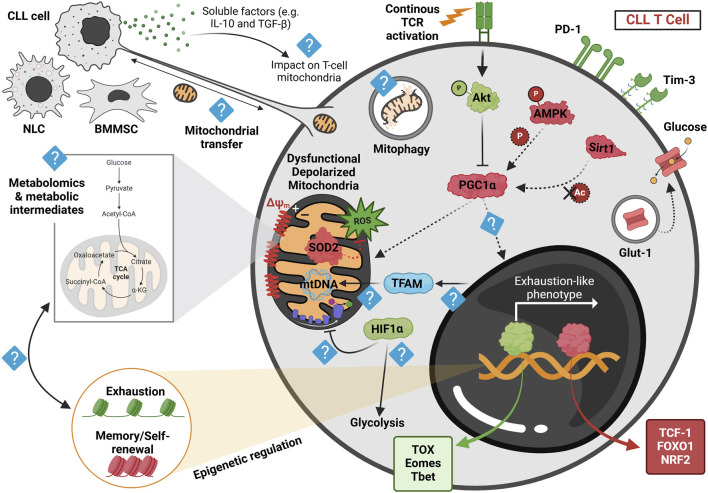
Proposed model of mitochondrial dysregulation in CLL-derived T cells at the basal state, highlighting current findings and future research directions. Human and murine CLL T cells accumulate depolarized mitochondria, leading to dysfunctional mitochondrial activity post *in vitro* activation. This depolarization correlates with elevated reactive oxygen species (ROS) levels, likely due to reduced expression of superoxide dismutase 2 (SOD2) observed in both human and murine cells. These mitochondrial disturbances, coupled with reduced levels of the transcription factor NRF2—an essential regulator of oxidative metabolism—are associated with the downregulation of PGC1α, the key regulator of mitochondrial function. Persistent stimulation of CLL T cells within the leukemic microenvironment is thought to activate Akt signaling, as recently demonstrated in resting CLL T cells from the adoptive transfer (AT) Eμ-TCL1 murine model, which may subsequently suppress PGC1α activity. Furthermore, recent findings suggest a downregulation of AMPK phosphorylation and lower Sirt1 expression in AT Eμ-TCL1 T cells, potentially impairing PGC1α function by limiting its phosphorylation and deacetylation, respectively. These metabolic alterations are also associated with decreased intracellular Glut-1 reserves in human CLL T cells, potentially hindering glucose uptake upon *in vitro* activation. Such metabolic disturbances correlate with an exhaustion-like T-cell differentiation phenotype, marked by increased expression of TOX, Eomes, and T-bet transcription factors, along with elevated PD-1 and Tim-3 levels, while displaying a diminished memory and stemness profile, as indicated by reduced expression of TCF-1 and increased FOXO1 phosphorylation. Despite these insights, key aspects of mitochondrial dysfunction in CLL T cells remain unclear. For instance, the influence of soluble factors such as IL-10 and TGF-β, released by CLL cells, on T-cell mitochondrial activity is unknown. Furthermore, the precise role of PGC1α in driving T-cell exhaustion remains to be investigated. Additionally, TFAM, a crucial mitochondrial transcription factor, warrants further study, particularly given recent findings that suggest reduced chromatin accessibility near TFAM in AT Eμ-TCL1 T cells. Studies in human CLL patient-derived T cells have indicated increased HIF1α expression; however, it remains to be determined whether this upregulation of hypoxic signature contributes to suppressed OXPHOS and enhanced glycolysis, as observed in solid tumors. Moreover, metabolomic analyses are needed to identify mitochondrial metabolite dysregulation in CLL-derived T cells and its relationship with the epigenetic changes driving exhaustion. Finally, exploring nanotube formation and mitochondrial transfer between T cells and microenvironmental cells may provide novel insights into CLL-associated metabolic dysregulation. Red represents downregulated proteins, green signifies upregulated proteins, and light blue indicates an unknown mechanism. Dashed arrows denote reduced regulation. NLC, nurse-like cell; BMMSC, bone marrow mesenchymal stromal cell. Figure was created in BioRender. Gamal et al. (2025), https://BioRender.com/lkbyyip.

The role of mitochondrial regulation in T-cell dysfunction has recently gained attention as a novel avenue for understanding immune dysregulation in CLL. Figure 2 visually summarizes current findings and potential future directions related to mitochondrial abnormalities in CLL T cells. As illustrated, T cells from CLL patients exhibit significant mitochondrial abnormalities in both CD8^+^ and CD4^+^ compartments, including excessive ROS production and reduced expression of the key mitochondrial regulator PGC1α and the ROS scavenging enzyme superoxide dismutase 2 (SOD2) (van Bruggen et al., 2019; van Bruggen et al., 2022). These mitochondrial disturbances are associated with diminished activation and proliferation of the CLL patients’ T cells and reduced glucose uptake capacity. Metabolic dysregulation in CLL T cells appears to be influenced by interactions with malignant CLL cells (van Bruggen et al., 2019; van Bruggen et al., 2022). This has been demonstrated *in vivo*, where CLL patients undergoing treatment with venetoclax and obinutuzumab exhibited enhanced CD4^+^ T-cell activation and increased glucose uptake compared to pre-treatment samples (van Bruggen et al., 2022). Furthermore, increased T-cell mitochondrial mass has been proposed as a potential biomarker for predicting response to CAR T-cell therapy in CLL patients (van Bruggen et al., 2019). This finding aligns with insights from a long-term follow-up study of two exceptional CLL cases in which patients sustained deep remission for over a decade following CAR T-cell therapy. In these cases, prolonged CAR T-cell persistence and expansion were specifically linked to enhanced glycolysis and mitochondrial OXPHOS, reinforcing the critical role of mitochondrial metabolism in therapeutic efficacy (Melenhorst et al., 2022).

The Eμ-TCL1 model is the most widely employed murine system for studying CLL, serving as a valuable tool for dissecting the disease’s microenvironment and immune interactions (Bichi et al., 2002). Recognizing its relevance, our group conducted a unique study integrating CLL patient samples with the Eμ-TCL1 model to explore T cells' metabolic regulation and differentiation dynamics in CLL (Gamal et al., 2025). This study validated the model’s utility in investigating T-cell mitochondrial dysfunction. Consistent with human data, we found that murine CLL CD8^+^ T cells exhibited heightened mitochondrial ROS levels. This was accompanied by reduced expression of PGC1α and SOD2 in a subset of T cells resembling an exhausted phenotype, characterized by elevated Eomes, TOX, PD-1, and Tim-3 expression, along with diminished TCF-1 levels. Functional mitochondrial assessment revealed a marked decline in mitochondrial SRC and a metabolic shift from OXPHOS to glycolysis following *in vitro* TCR stimulation in both human and murine CLL CD8^+^ T cells. Additionally, mitochondrial phenotyping demonstrated an accumulation of depolarized mitochondria with decreased membrane potential relative to mitochondrial mass in resting CD8^+^ T cells from both patient samples and the Eμ-TCL1 model (Gamal et al., 2025).

Multi-omics profiling of the transcriptome and epigenome of murine CLL T cells throughout disease progression unveiled significant disruptions in mitochondrial signaling. These included reduced chromatin accessibility near mitochondrial-associated genes (*Tfam*, *Vdac1*, *Timm8a2*), suppressed AMP-activated protein kinase (AMPK) and autophagy activity, and increased glycolysis at late disease stages (Gamal et al., 2025). Notably, AMPK plays a crucial role in metabolic stress responses, mitochondrial biogenesis (Herzig and Shaw, 2018), and mitophagy (Laker et al., 2017; Hung et al., 2021). These metabolic alterations correlated with the increase of an exhausted-like T-cell phenotype in late-stage CLL (Gamal et al., 2025).

These findings underscore the intricate role of mitochondrial dysfunction in driving T-cell impairment in CLL. However, further mechanistic studies are necessary to unravel the precise molecular pathways underlying this process.

## Reprogramming T-cell mitochondrial activity as a promising therapeutic approach

Given the pivotal role of mitochondrial dysfunction in T-cell impairment, recent research has focused on innovative strategies to rewire mitochondrial metabolism in T cells. These approaches aim to improve T-cell function both *in vivo* and in the context of adoptive cell transfer, ultimately enhancing cancer immunotherapy outcomes. The ideal immune cell for ACT exhibits self-renewal and pluripotency, attributes that address the current limitations of ACT. Enhancing mitochondrial function to bolster T-cell memory represents a key objective in this approach. Notably, PGC1α has emerged as a crucial regulator of mitochondrial biogenesis, fusion, and antioxidant activity, making it an attractive target for improving T-cell fitness in cancer (Finck and Kelly, 2006; Gureev et al., 2019). Overexpression of PGC1α in T cells has enhanced mitochondrial activity and biogenesis, fostered a memory-like phenotype, and improved tumor eradication in murine melanoma models (Scharping et al., 2016; Dumauthioz et al., 2021). More recently, this strategy has been extended to engineering human CAR T cells for solid tumors, yielding promising results with increased mitochondrial fitness and enhanced tumor clearance, correlating with a more stem-like phenotype (Lontos et al., 2023).

PGC1α regulation is dynamically influenced by several signaling pathways relevant to T-cell activation, with the PI3K/Akt axis playing a prominent inhibitory role (Fernandez-Marcos and Auwerx, 2011). Elevated Akt signaling has been strongly associated with PGC1α downregulation and mitochondrial dysfunction in TILs (Scharping et al., 2016). In the context of CLL, our recent findings demonstrate that heightened basal Akt signaling correlates with mitochondrial impairment in Eμ-TCL1 T cells (Gamal et al., 2025). Thus, targeting the PI3K/Akt pathway offers a compelling approach for mitochondrial reprogramming of T cells (Rangel Rivera et al., 2023). *Ex vivo* inhibition of PI3Kδ using idelalisib during the culture of human or murine CLL T cells has increased central memory T-cell frequency and downregulated exhaustion markers. Additionally, it reprogrammed T-cell metabolism by enhancing mitochondrial SRC and maximal mitochondrial capacity relative to glycolytic activity (Gamal et al., 2025). In murine experiments, *ex vivo* idelalisib priming of Eμ-TCL1-derived splenic T cells enhanced mitochondrial membrane potential and SRC while increasing PGC1α expression (Gamal et al., 2025). This treatment also upregulated AMPKα phosphorylation and Sirt1 expression, which are known to promote PGC1α-mediated mitochondrial enhancement and memory T-cell formation (Ma et al., 2017). Additionally, idelalisib fostered mitochondrial fusion (Gamal et al., 2025), suggesting a shift of Eμ-TCL1 T cells toward a central memory fate (Buck Michael et al., 2016). Translationally, *ex vivo* preconditioning of Eμ-TCL1 CAR T cells with idelalisib resulted in significant anti-leukemic effects and prolonged survival following infusion in CLL-bearing immunocompetent mice (Gamal et al., 2025). Similarly, treatment of human CLL CAR T cells with the PI3Kδ/γ inhibitor duvelisib augmented mitochondrial content and fusion, enhancing memory properties and improving *in vivo* outcomes in NOG mice engrafted with human CLL cell line (Funk et al., 2022). Consistent with the beneficial impact of PI3K/Akt inhibition on T-cell mitochondrial function, recent studies have shown that overexpression of FOXO1, a downstream target of Akt, fosters a stem-like phenotype in CAR T cells. This effect has been observed in CAR T cells derived from healthy donors and patients. This stem-like phenotype was associated with enhanced mitochondrial fitness, increased OXPHOS, more remarkable persistence, and improved *in vivo* therapeutic activity (Chan et al., 2024; Doan et al., 2024).

Excessive mitochondrial ROS production contributes to T-cell exhaustion by impairing ATP synthesis via iron-sulfur cluster inactivation in the ETC (Vardhana et al., 2020). Mitigating mitochondrial ROS has emerged as a promising intervention to restore effector T-cell function in cancer and chronic infections. For instance, supplementation with the mitochondria-targeted antioxidant MitoTempo has been shown to prevent mitochondrial depolarization in T cells post-activation (Yu et al., 2020). Additionally, N-acetyl cysteine (NAC) treatment during chronic T-cell stimulation increased mitochondrial oxygen consumption, rescued ATP production, enhanced proliferation, and shifted gene expression from terminal exhaustion to a progenitor-like state (Vardhana et al., 2020). In preclinical studies, adoptive transfer of NAC-treated OT-I T cells improved survival in melanoma-bearing mice receiving anti-PD-L1 therapy (Vardhana et al., 2020). Similarly, activation of human renal cell carcinoma TILs in the presence of ROS scavengers such as MitoQ or MitoTempo enhanced TIL activation (Siska et al., 2017), further supporting the importance of mitochondrial redox balance in T-cell functionality.

Reinforcing the significance of mitochondrial fitness in T-cell survival and function, studies have shown that isolating T cells with low mitochondrial membrane potential enriches metabolically resilient populations. These cells exhibit enhanced persistence and improved tumor-eradicating capacity (Sukumar et al., 2016). Moreover, recent advancements in T-cell metabolism research highlight the potential of modulating metabolic intermediates as a promising strategy for reprogramming T-cell activity to enhance cancer immunotherapy (Liu et al., 2022). Pharmacological screening of key mitochondrial components has identified isocitrate dehydrogenase 2 (IDH2) as a critical regulator that hinders metabolic adaptation necessary for optimal memory CAR T-cell formation and functionality (Si et al., 2024). Inhibiting IDH2 pharmacologically or genetically redirected glucose metabolism toward the pentose phosphate pathway, enhancing antioxidant capacity and reducing T-cell exhaustion. Additionally, IDH2 inhibition diverted citrate into the cytosol, supporting acetyl-coenzyme A-mediated epigenetic modifications that promote memory differentiation of CAR T cells (Si et al., 2024). Similarly, targeting the mitochondrial pyruvate carrier (MPC) during CAR T-cell manufacturing has been shown to improve the memory phenotype by promoting metabolic flexibility through alternative acetyl-coenzyme A production pathways, thereby enhancing histone acetylation and pro-memory gene expression (Wenes et al., 2022). This metabolic intervention has demonstrated durable antitumor efficacy in solid tumor and leukemia models (Wenes et al., 2022).

Beyond intrinsic mitochondrial programming, intercellular mitochondrial transfer between cancer cells and T cells has emerged as a novel immune evasion mechanism (Saha et al., 2022; Zhang et al., 2023; Ikeda et al., 2025). Baldwin et al. leveraged this concept to enhance ACT efficacy by using bone marrow stromal cells (BMSCs) to transfer mitochondria to CD8^+^ T cells, significantly augmenting their metabolic fitness and antitumor activity (Baldwin et al., 2024). This approach was effective across multiple T-cell platforms (TCR, CAR, and TILs) both *in vitro* and *in vivo*. Mitochondrial transfer from BMSCs markedly enhanced T-cell mitochondrial oxygen consumption, SRC, persistence, tumor infiltration, and resistance to exhaustion, culminating in improved cytotoxic function (Baldwin et al., 2024). Furthermore, targeting Ras/Rho GTPase-mediated intercellular mitochondrial transfer using L-778123, a dual farnesyltransferase and geranylgeranyltransferase inhibitor, significantly reduced mitochondrial hijacking by cancer cells (Saha et al., 2022). In a syngeneic breast tumor model, combining L-778123 with PD-1 blockade resulted in superior tumor control and increased CD8^+^ T-cell infiltration (Saha et al., 2022). This suggests that inhibiting mitochondrial transfer may represent a novel avenue to improve cancer immunotherapy.

Taken together, mitochondrial metabolism profoundly impacts T-cell differentiation and function, making the modulation of key metabolic intermediates a promising strategy for reprogramming T-cell activity to enhance immunotherapeutic efficacy. By leveraging insights into mitochondrial biogenesis, oxidative metabolism, and intercellular mitochondrial dynamics, future research can further refine metabolic interventions to optimize T-cell-based cancer therapies.

## Discussion and future research directions

T-cell dysfunction in CLL has been recognized for several years, with current significant advancements in defining T-cell differentiation phenotypes, transcriptional and epigenetic signatures (Gamal et al., 2025; Hanna et al., 2019; Hanna et al., 2021; Llaó-Cid et al., 2021). However, emerging research has expanded our understanding of T-cell exhaustion by highlighting cellular organelles' critical role in shaping immune dysfunction, particularly mitochondria. This review explored how T-cell mitochondrial metabolism and quality are disrupted within the metabolically hostile TME, contributing to T-cell exhaustion and impaired anti-tumor immunity.

Despite the growing recognition of T-cell mitochondrial dysfunction in CLL, key questions remain unanswered. Addressing these questions could enhance our understanding and inform potential strategies for targeting T-cell dysfunction in CLL. Some of these uncertainties are visually highlighted in Figure 2. For example, it has been shown that CLL T cells exhibit reduced glucose uptake as a result of indirect contact with CLL cells, as demonstrated by trans-well experiments (van Bruggen et al., 2019). However, it remains unclear which soluble factors released by CLL cells can induce this effect and whether these factors also contribute to the mitochondrial dysfunction in T cells. *In vitro* experiments incorporating conditioned-culture media from CLL cells and measuring T-cell mitochondrial phenotype and activity could provide further insights about these soluble factors.

The leukemic microenvironment plays a significant role in CLL-cell proliferation *in vivo*. NLCs, a type of CLL-specific tumor-associated macrophage-like cells, are known to support CLL-cell survival (Koehrer and Burger, 2023). Conducting *in vitro* experiments to generate NLCs from CLL patient samples and co-culturing them with T cells, either in the presence or absence of CLL cells, could help determine their role in T-cell mitochondrial disruption. Additionally, research has demonstrated that the lymph node microenvironment in CLL patients exerts greater immunosuppressive effects on T cells than peripheral blood (de Weerdt et al., 2019; Hanna et al., 2019). Recently, novel 3D *in vitro* culture systems have been developed to mimic the lymph node microenvironment in CLL patients (Haselager et al., 2023; Playà-Albinyana et al., 2024). These models could be used to study the impact of the CLL microenvironment on T-cell mitochondrial activity from a clinical perspective. Additionally, these novel culture systems can also be valuable for studying the role of intercellular nanotube-mediated mitochondrial transfer in T-cell dysfunction within the context of the CLL microenvironment.

Metabolomic studies should be emphasized to elucidate the role of various mitochondrial intermediates and their potential involvement in the epigenetic regulation of T-cell differentiation in CLL. Combining metabolomic analyses with isotope-labeled intermediate tracing experiments in CLL T cells could provide a more complete picture of the disrupted mitochondrial/metabolic pathways. Additionally, since antigen-specific stimulation is thought to contribute to T-cell dysfunction in CLL (Hanna et al., 2019; Vardi et al., 2016; Vardi et al., 2017; Blanco et al., 2018; Serrano et al., 1997; Kowalewski et al., 2015), more studies using single-cell RNA and TCR sequencing could help explore mitochondrial disturbances in specific CLL T-cell clones in patients.

A key unanswered question in the field is whether mitochondrial abnormalities in T cells are a primary driver of dysfunction or a secondary consequence of chronic antigenic stimulation and immune suppression. Understanding these mechanistic underpinnings will be essential for identifying novel therapeutic targets to restore T-cell function. The Eμ-TCL1 murine model of CLL offers a valuable platform for mechanistic studies to delineate the precise molecular pathways governing mitochondrial metabolism in T cells. For instance, crossing Eμ-TCL1 mice with T-cell-specific knockout models for essential mitochondrial molecules, such as the mitochondrial phosphate carrier (mPiC), which is crucial for ATP production and OXPHOS (Wu et al., 2023), could help determine the causal relationship between mitochondrial dysfunction and T-cell exhaustion in CLL.

TFAM, a key regulator of mitochondrial DNA transcription, replication and maintenance, and PGC1α, a master regulator of mitochondrial biogenesis, are dysregulated on the epigenetic and protein levels respectively in CLL T cells (Gamal et al., 2025; van Bruggen et al., 2019). Using the Eμ-TCL1 model to study knockout or overexpression of these molecules specifically in T cells would provide crucial mechanistic insights into T-cell mitochondrial disturbances in CLL. Additionally, increased HIF1α levels in resting CLL patients’ T cells (van Bruggen et al., 2019) and a pseudohypoxic state following *in vitro* activation with CLL cells (Montironi et al., 2023) contribute to T-cell dysfunction. Further research is needed to understand how HIF1α regulates mitochondrial fitness and glycolytic activity in CLL T cells, particularly given recent findings linking mitochondrial dysfunction to T-cell exhaustion via HIF1α-mediated glycolytic reprogramming (Wu et al., 2023).

T-cell exhaustion is a complex, multifactorial process involving cellular and metabolic alterations and epigenetic reprogramming. Epigenetic modifications play a crucial role in establishing the long-lasting gene expression profiles that define T-cell exhaustion (Abdel-Hakeem et al., 2021; Huang et al., 2025). Mitochondrial dysfunction can impact these epigenetic processes by generating metabolic signals that alter the activity of epigenetic modifiers. For instance, changes in NAD+ levels due to impaired mitochondrial function can influence sirtuin activity, enzymes that regulate histone deacetylation and chromatin remodeling (Hamaidi and Kim, 2022). Similarly, mitochondrial-derived ROS can act as signaling molecules that modulate DNA methyltransferase activity (Al-Awar and Hussain, 2024), which might lead to changes in DNA methylation promoting T-cell exhaustion. Given the intricate relationship between mitochondrial metabolism and epigenetic regulation (Franco et al., 2020), examining how these two factors interact in CLL T cells could yield critical insights into the mechanisms of T-cell exhaustion. The Eμ-TCL1 murine model offers an ideal platform for studying these connections *in vivo*, which could open new avenues for therapeutic interventions to reverse exhaustion and restore T-cell function in CLL.

Recent studies have highlighted that CLL T-cell dysfunction is closely associated with impaired lipid metabolism (Jacobs et al., 2025). This involves key alterations including a reduction in exogenous cholesterol uptake, a compromised ability to initiate *de novo* fatty acid synthesis, decreased FAO, a lower relative abundance of cholesterol and phospholipids, and an accumulation of triglycerides. Using the Eμ-TCL1 murine model to understand how these metabolic disturbances interact with mitochondrial dysfunction and contribute to T-cell exhaustion in CLL could provide important insights into potential therapeutic targets.

Pharmacological interventions targeting mitochondrial dysfunction represent another promising avenue for future research. Understanding the role of mitochondrial ROS in CLL T-cell dysfunction could be explored using mitochondrial antioxidants such as MitoTempo, MitoQ, or NAC during *in vitro* activation of CLL T cells. Additionally, evidence suggests the involvement of dysregulated oxidative stress responses, including AMPK signaling and autophagy, in CLL T-cell dysfunction (Gamal et al., 2025). Targeting these pathways with AMPK agonists like AICAR or mitophagy inducers like nicotinamide riboside (NR) could provide novel therapeutic insights. Mitochondrial dynamics also play a crucial role in T-cell function, with mitochondrial fission and fusion contributing to T-cell activation and persistence, respectively (Buck Michael et al., 2016; Baixauli et al., 2011). The effects of pharmacological modifiers such as Mdivi-1, a Drp1 inhibitor that can reduce mitochondrial fission, should be explored in murine and human CLL T cells.

From a clinical perspective, targeting mitochondrial metabolism to improve CAR T-cell therapy outcomes in CLL is critical. Fraietta et al. demonstrated using transcriptional analysis that complete response in CLL patients receiving CD19-specific CAR T cells with 4-1BB/CD3ζ domains is correlated with reduced glycolytic signature and increased memory-like phenotype of the infused CAR T cells (Fraietta et al., 2018). van Bruggen et al. analyzed mitochondrial readouts in CD8^+^ CAR^+^ T cells from the infusion products of 27 relapsed/refractory CLL patients in the trials NCT01029366 and NCT01747486 (van Bruggen et al., 2019). They found that while glucose uptake, mitochondrial membrane potential, and mitochondrial ROS were similar between complete responders (CRs) and non-responders (NRs), mitochondrial mass was significantly higher in CRs. Mitochondrial mass correlated with CAR T-cell persistence markers, including *in vitro* expansion, peak expansion (days 0–35), and CD3^+^CD8^+^ cell percentage in the first 28 days post infusion. It also correlated with non-exhausted CD27^+^ T cells lacking inhibitory receptors (PD-1, Tim-3, LAG-3). Additionally, single-cell multiomic analysis of peripheral blood samples from two CLL patients who received CD19-specific CAR T cells with 4-1BB/CD3ζ domains and achieved complete remission showed that durable responses (∼10 years) were linked to clonotypic expansion of CD4^+^ CAR T cells (Melenhorst et al., 2022). These cells showed signs of *in vitro* and *in vivo* activation with upregulated transcriptional signature of antigen mediated and TCR signaling. Interestingly, glycolytic transcriptional signature indicated by *GAPDH* was upregulated across all cell-cycle phases of these surviving CAR T cells, while OXPHOS and mitochondrial protein complex pathways were elevated in actively cycling cells (G2/M and S phases), highlighting the role of mitochondria in CAR T-cell survival in CLL.

These findings suggest that rewiring mitochondrial metabolism could improve CAR T-cell therapy in CLL. Pharmacological inhibitors, such as PI3K inhibitors, are promising tools to enhance T-cell mitochondrial activity and memory differentiation and improve CAR T-cell function in CLL (Gamal et al., 2025; Funk et al., 2022). However, gene editing technologies such as CRISPR/Cas9 are emerging as powerful tools for introducing permanent modifications in CAR T cells for clinical use (Tao et al., 2024), offering more durable improvements compared to the transient effects of small-molecule inhibitors. Moreover, genetic approaches can target previously undruggable targets, presenting promising new avenues for enhancing CAR T-cell therapy in CLL. Although not CRISPR/Cas9-based, viral transduction was successfully used by Roselle et al. to generate Δ133p53α (an endogenous isoform of the human TP53 gene)-expressing CAR T cells from non-responding CLL patients in trial NCT01029366 (Roselle et al., 2024). This modification enhanced *in vitro* tumor-killing capacity, increased mitochondrial SRC, and improved efficacy in a xenograft mouse model.

## Conclusion

In conclusion, our review systematically examined the role of mitochondrial dysregulation in CLL T-cell dysfunction, shedding light on key mechanistic insights and identifying critical research gaps. We outlined future directions to deepen our understanding and uncover novel mitochondrial-related therapeutic targets. Given the persistently low success rates of CAR T-cell therapy in CLL, there is an urgent need to integrate mitochondrial-targeting approaches to enhance CAR T-cell persistence and function.

Future research must bridge the gap between mitochondrial biology and immunotherapy to drive meaningful clinical advancements, leveraging cutting-edge approaches such as gene editing, metabolic reprogramming, and pharmacological modulation. By harnessing these strategies, we can overcome immune suppression in CLL and improve the effectiveness of CAR T-cell therapy, bringing better outcomes for patients.
